# Pharmacokinetics and safety of a single dose of telavancin in pediatric subjects 2–17 years of age

**DOI:** 10.1128/aac.00987-23

**Published:** 2023-10-10

**Authors:** John S. Bradley, Jennifer L. Goldman, Laura P. James, Byron Kaelin, Breanne H. Y. Gibson, Antonio Arrieta

**Affiliations:** 1 Department of Pediatrics, University of California San Diego School of Medicine and Rady Children’s Hospital, San Diego, California, USA; 2 Department of Pediatrics, University of Missouri-Kansas City and Children’s Mercy Hospital, Kansas City, Missouri, USA; 3 Department of Pediatrics, Arkansas Children’s Hospital Research Institute, Little Rock, Arkansas, USA; 4 Product Development, Cumberland Pharmaceuticals Inc., Nashville, Tennessee, USA; 5 Division of Infectious Diseases, Children’s Hospital of Orange County, Orange County, California, USA; Providence Portland Medical Center, Portland, Oregon, USA

**Keywords:** antimicrobial agents, clinical therapeutics, pharmacokinetics, pediatric drug therapy

## Abstract

Antimicrobial resistance increases infection morbidity in both adults and children, necessitating the development of new therapeutic options. Telavancin, an antibiotic approved in the United States for certain bacterial infections in adults, has not been examined in pediatric patients. The objectives of this study were to evaluate the short-term safety and pharmacokinetics (PK) of a single intravenous infusion of telavancin in pediatric patients. Single-dose safety and PK of 10 mg/kg telavancin was investigated in pediatric subjects >12 months to ≤17 years of age with known or suspected bacterial infection. Plasma was collected up to 24-h post-infusion and analyzed for concentrations of telavancin and its metabolite for noncompartmental PK analysis. Safety was monitored by physical exams, vital signs, laboratory values, and adverse events following telavancin administration. Twenty-two subjects were enrolled: 14 subjects in Cohort 1 (12–17 years), 7 subjects in Cohort 2 (6–11 years), and 1 subject in Cohort 3 (2–5 years). A single dose of telavancin was well-tolerated in all pediatric age cohorts without clinically significant effects. All age groups exhibited increased clearance of telavancin and reduced exposure to telavancin compared to adults, with mean peak plasma concentrations of 58.3 µg/mL (Cohort 1), 60.1 µg/mL (Cohort 2), and 53.1 µg/mL (Cohort 3). A 10 mg/kg dose of telavancin was well tolerated in pediatric subjects. Telavancin exposure was lower in pediatric subjects compared to adult subjects. Further studies are needed to determine the dose required in phase 3 clinical trials in pediatrics.

## INTRODUCTION

Skin, respiratory, and urinary tract bacterial infections are common causes of hospitalization in pediatric populations, and the rise of antimicrobial resistance (AMR) impacts the effectiveness of empiric and definitive treatment, increasing overall morbidity (1 – 3). In 2019, there were an estimated 1.27 million deaths globally due to AMR bacterial infection, with methicillin-resistant *Staphylococcus aureus* (MRSA) alone responsible for 100,000 infection-related deaths in both adults and children (2, 4, 5). Antibiotic failure is a significant challenge in the treatment of infections, the major causes of which are AMR, the presence of biofilms, sepsis, and compromised immunity (6). While early and effective treatment is critical in the prevention of sepsis-related antibiotic failure, a critical solution to antibiotic failure has been finding new antibiotics to address AMR and biofilm-resident pathogens (6). In children, antibiotic options are increasing for MRSA, but a need still exists for more safe and effective agents with well-characterized pediatric and neonatal pharmacokinetics (PK) (2, 3).

Telavancin is a novel lipoglycopeptide antibiotic with both glycopeptide activity characteristic of vancomycin, and lipopeptide activity characteristic of daptomycin. Telavancin has demonstrated bactericidal efficacy against Gram-positive pathogens, including MRSA, vancomycin-intermediate *S. aureus* (VISA), and other linezolid-resistant, and daptomycin-nonsusceptible bacterial strains (7 – 9). Telavancin is approved for the treatment of complicated skin and skin structure infections (cSSSIs) and hospital-acquired or ventilator-associated bacterial pneumonia (HABP/VABP) in adults (10 – 12) and has also demonstrated positive clinical outcomes in patients with bone and joint infections, bacteremia, and endocarditis caused by Gram-positive bacteria resistant to standard-of-care antibiotic therapies (13 – 15).

To date, telavancin has not been thoroughly examined in pediatric subjects. While limited case studies have highlighted the use of telavancin in pediatric cystic fibrosis patients >12 years of age and >45 kg (16), no prospective clinical trials have evaluated the PK and safety of telavancin in a pediatric population. The objectives of this study were to evaluate both the PK and the short-term safety of a single 10 mg/kg dose of telavancin in pediatric patients >12 months of age.

## RESULTS

### Study subjects

Between 14 July 2015 and 29 October 2018, a total of 22 subjects were enrolled across seven clinical trial sites in the United States. The conditions for which subjects required systemic antibiotic therapy are summarized in Table 1. All 22 subjects received an intravenous (IV) infusion of 10 mg/kg telavancin on Day 1 of the study. Twenty-one subjects completed the study through the Day 8 (±1 day) safety follow-up call, and one subject was discharged and lost to follow-up prior to completion of all study procedures.

**TABLE 1:** Conditions requiring systemic antibiotic therapy by cohort

Condition	Cohort 1 (12–17 years) *N* = 14	Cohort 2 (6–11 years) *N* = 7	Cohort 3 (2–5 years) *N* = 1
Bacteremia	0	1	1
Bone/joint infection	1	2	0
Gastrointestinal tract perforation^*a*^	3	1	0
Skin/soft tissue infection	3	1	0
Post-operative infection^*b*^	3	1	0
Respiratory tract infection	3	1	0
Unspecified^*c*^	1	0	0

^*a*^ Includes perforated/ruptured appendicitis.

^*b*^ Includes both treatment and prevention.

^*c*^ Subject hospitalized with a medical history of cystic fibrosis.

### Subject demographics

There were 14 subjects enrolled in Cohort 1 (12–17 years), 7 subjects enrolled in Cohort 2 (6–11 years), and 1 subject in Cohort 3 (2–5 years). There were no subjects enrolled in Cohort 4 (1 to <2 years). Cohort 1 enrolled beyond its target enrollment of eight subjects due to several subjects missing post-dose safety laboratory measurements. The youngest subject enrolled was 2 years of age, and the oldest subject enrolled was 17 years of age. The median ages for Cohorts 1, 2, and 3 were 15.5, 10.0, and 2.0 years, respectively. Of all subjects enrolled, 1 subject (4.5%) was Black and non-Hispanic or Latino, 9 subjects (40.9%) were White and Hispanic or Latino, 11 subjects (50.0%) were White and non-Hispanic or Latino, and 1 subject was White with no additional information reported. Ten subjects (45.5%) were females, and 12 subjects (54.5%) were males. The median weights for Cohorts 1, 2, and 3 were 61.0, 35.4g, and 12.3 kg, respectively. Subject demographics are summarized by cohort in Table 2.

**TABLE 2:** Demographic characteristics of treated subjects by age cohort (*N* = 22)

Characteristic^*a*^	Cohort 1 (12–17 years)	Cohort 2 (6–11 years)	Cohort 3 (2–5 years)	All subjects
Number of subjects	14	7	1	22
Age (years)	15.5 (12.0–17.0)	10.0 (6.0–11.0)	2.0	14.0 (2.0–17.0)
BMI (kg/m^2^)	23.1 (18.1–28.8)	18.8 (15.9–21.2)	18.7	20.9 (15.9–28.8)
Height (cm)	165 (145–183)	137 (115–151)	81.0	156 (81.0–183)
Weight (kg)	61.0 (43.7–89.1)	35.4 (21.0–48.3)	12.3	48.4 (12.3–89.1)
Sex, *N* (%)				
Female	8 (57.1)	1 (14.3)	1 (100)	10 (45.5)
Male	6 (42.9)	6 (85.7)	0 (0)	12 (54.5)
Ethnicity/genetic background, *N* (%)				
Black	1 (7.1)	0 (0)	0 (0)	1 (4.5)
White, Hispanic, or Latino	4 (28.6)	4 (57.1)	1 (100)	9 (40.9)
White, Non-Hispanic, or Latino	8 (57.1)	3 (42.9)	0 (0)	11 (50.0)
White, not reported	1 (7.14)	0 (0)	0 (0)	1 (4.55)

^*a*^ Characteristics described as median (minimum–maximum) unless otherwise specified; BMI = body mass index; cm = centimeters; kg = kilograms; m = meters; *N* = number of subjects.

### Pharmacokinetic results

**Fig 1:**
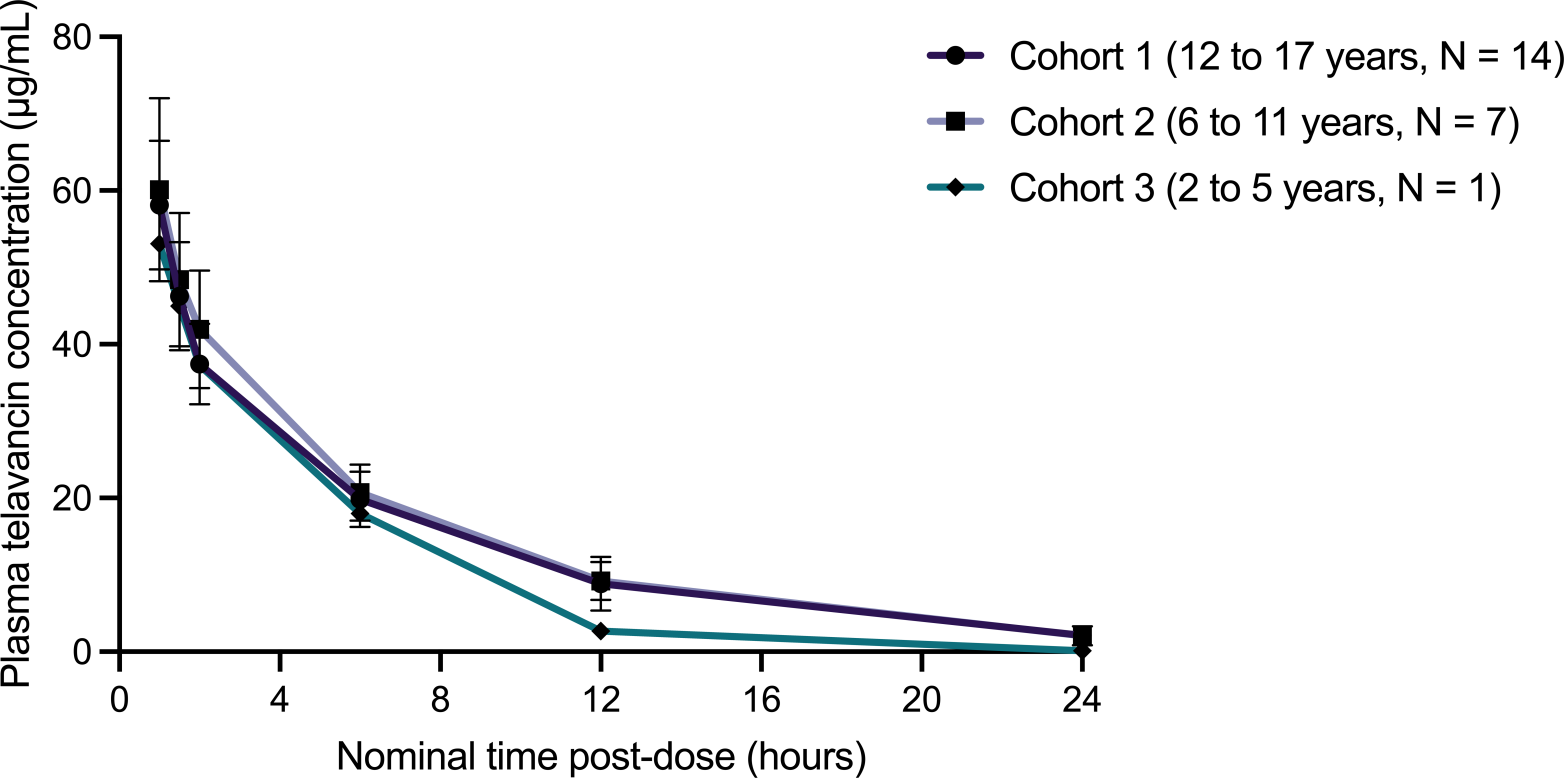
Plasma telavancin concentrations over 24 h following a single 10 mg/kg IV infusion of telavancin by age cohort. Values are expressed as the mean concentration for each time point; error bars represent the standard deviation.

**Fig 2:**
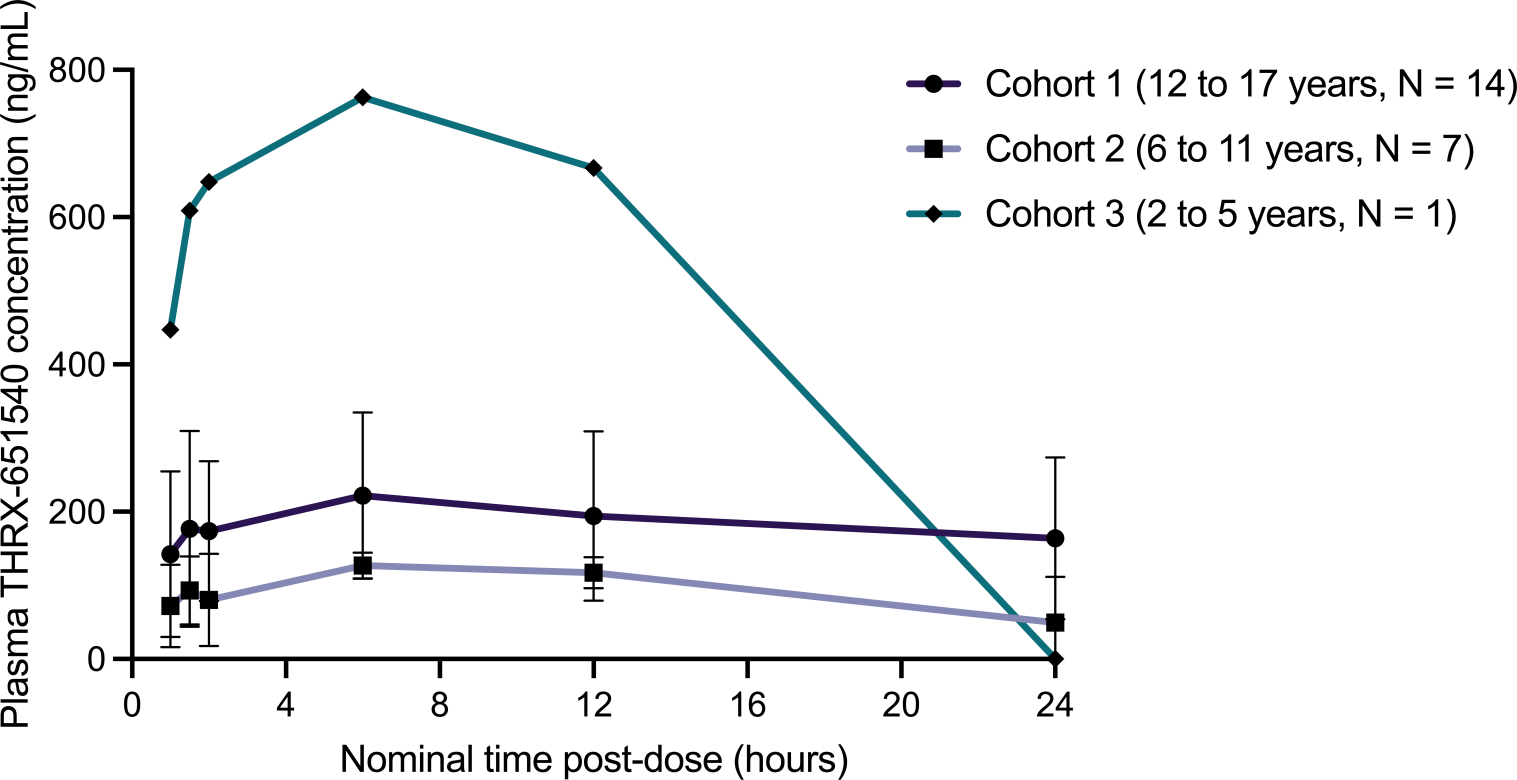
Plasma THRX-651540 concentrations following a single 10 mg/kg IV infusion of telavancin by age cohort. Values are expressed as the mean concentration for each time point; error bars represent the standard deviation.

PK analysis was performed from the individual plasma concentration values of both telavancin and its primary metabolite, THRX-651540. All 22 enrolled subjects were included in the PK population, and 20 subjects were included in the PK analysis for modeling. One subject in Cohort 1 (12–17 years of age) and one subject in Cohort 2 (6–11 years of age) were not included in the PK analysis due to lack of PK samples at early time points. Mean plasma telavancin concentrations at each collected time point are summarized in Table 3, and plasma telavancin and THRX-651540 concentrations over time are displayed in Fig. 1 and Fig. 2, respectively. Plasma concentration profiles for telavancin were similar between Cohorts 1 and 2, and one subject in Cohort 3 exhibited reduced exposure to telavancin (Fig. 1). For telavancin, the mean *C*_max_ for Cohorts 1, 2, and 3 was 58.3, 60.1, and 53.1 µg/mL, respectively. The mean AUC_0-inf_ for Cohorts 1, 2, and 3 was 345, 351, and 229 h*μg/mL, respectively, which is lower than the mean AUC_0-inf_ of 747 h*μg/mL observed following a single 10 mg/kg IV dose of telavancin in healthy adults (Table 4)(12). The mean *T*_1/2_ was lower in pediatric subjects at 5.60, 5.19, and 2.72 h for Cohorts 1, 2, and 3, respectively, compared with the mean *T*_1/2_ of 8.0 h found in healthy adult subjects (12). Plasma concentration profiles of THRX-651540 were similar between Cohorts 1 and 2, with slightly higher concentrations in Cohort 1. While PK analysis was not conducted for the metabolite, plasma concentrations of THRX-651540 in children 6–17 years were lower compared to those previously reported in healthy adults (17). The single subject in Cohort 3 exhibited elevated plasma THRX-651540 levels compared with both Cohorts 1 and 2 (Fig. 2) and with previous findings in healthy adults (17).

**TABLE 3:** Mean plasma telavancin concentrations at each time point^*a*^

Nominal time (h)	Cohort 1 (12–17 years) *N* = 13–14	Cohort 2 (6–11 years) *N* = 6–7	Cohort 3 (2–5 years) *N* = 1
1	58.1 ± 8.38	60.1 ± 11.9	53.1
1.5	46.2 ± 7.01	48.4 ± 8.66	45.0
2	37.4 ± 5.26	41.9 ± 7.65	37.3
6	19.8 ± 3.58	20.7 ± 3.64	18.0
12	8.84 ± 3.51	9.20 ± 2.50	2.67
24	2.14 ± 0.922	2.08 ± 1.23	0.146

^*a*^ All values represented as mean ± standard deviation; *N* = number of subjects.

**TABLE 4:** Pharmacokinetic parameters in pediatric subjects and in healthy adult subjects

PK parameter^*a*^	Cohort 1 *N* = 13	Cohort 2 *N* = 6	Cohort 3 *N* = 1	Healthy adults (12) *N* = 42
*C*_*max*_ *(*μg/mL*)*	58.3 ± 8.40	60.1 ± 11.9	53.1	93.6 ± 14.2
*T*_max^*b*^_ (hours)	1.02 (1.00–1.55)	1.09 (1.00–1.32)	1.18	NM
AUC_0-inf_ (h*μg/mL)	345 ± 58.5	351 ± 79.7	229	747 ± 129
AUC_0-last_^c^ (h*μg/mL)	326 ± 50.0	333 ± 74.2	228	666 ± 107^c^
*T*_1/2_ (h)	5.60 ± 1.01	5.19 ± 0.751	2.73	8.0 ± 1.5
CL (mL/h/kg)	29.8 ± 5.5	30.0 ± 8.1	43.7	13.9 ± 2.9
Vss (mL/kg)	218 ± 25.5	204 ± 36.2	173	145 ± 23

^*a*^ All values reported as mean ± standard deviation unless otherwise noted^.^

^*b*^ medi (minimum–maximum); AUC_0-inf_ = area under the curve extrapolated to infinity; AUC_0-last_ = area under the curve from time zero until the last PK sample; CL = clearance; *C*_max_ = maximum plasma concentration; *N* = number of subjects; NM = not measured; *T*_max_ = time at maximum concentration; *T*_1/2_ = terminal elimination half-life; V_ss_ = apparent steady state volume of distribution.

### Safety results

During the study period, a total of 27 adverse events (AEs) occurred in 10 of the 22 subjects (45%). In Cohort 1, 16 AEs occurred across six patients. Of the 16 AEs in Cohort 1, 13 were mild and 3 were moderate in severity. Fifteen of the 16 events (94%) that occurred in Cohort 1 were deemed probably related to telavancin administration. In Cohort 2, 11 AEs occurred across four patients; of the 11 AEs, nine were mild and two were moderate in severity. Of the 11 events that occurred in Cohort 2, six (55%) were considered probably related to telavancin administration. No AEs were reported for the single subject in Cohort 3. The most common AEs reported across all cohorts were nausea (four subjects, 18%), taste disturbance/metallic taste (three subjects, 14%), foamy urine (three subjects, 14%), dizziness (two subjects, 9%), and headache (two subjects, 9%). There were no serious AEs reported during this study. Of the safety laboratory measures collected at screening and on Day 2, there were three clinically significant abnormalities following telavancin administration. One subject in Cohort 1 (12–17 years) admitted for right lobe pneumonia with previously elevated liver enzymes experienced both worsening of elevated alanine aminotransferase and worsening of elevated lactate dehydrogenase following telavancin administration. The subject was treated with amoxicillin, azithromycin, and ondansetron prior to telavancin administration and received ceftriaxone, ibuprofen, and albuterol during the telavancin treatment period. One subject in Cohort 2 (6–11 years of age) experienced a prolonged QT interval following telavancin administration. These events were considered moderate in severity and resolved prior to Day 8. With the exception of the aforementioned laboratory abnormalities, no safety signals were identified from blood laboratory markers, including blood count, serum creatinine, and liver enzymes. A brief summary of select pre- and post-dose laboratory values by cohort is displayed in Table S1.

## DISCUSSION

Telavancin, a rapidly bactericidal antibiotic with concentration-dependent activity against Gram-positive pathogens, is Food and Drug Administration (FDA)-approved to treat specific infections in adults (12), but it has not been prospectively investigated in pediatric patients. In this open-label study evaluating the PK and short-term safety of telavancin following a single 10 mg/kg dose in pediatric subjects aged 2–17 years, exposure to telavancin was lower than that reported in healthy adults (12, 18, 19). The terminal elimination half-life of telavancin was shorter in pediatric subjects than in adults, due to an expected increase in renal clearance observed in pediatric patients, similar to that noted with glycopeptide antibiotics (20, 21). The one subject in Cohort 3 (2–5 years) exhibited reduced exposure to telavancin and increased exposure to the THRX-651540 metabolite; however, this child had a medical history of trichohepatoenteric syndrome with possible alterations in volume of distribution, metabolism, and clearance. One subject in Cohort 1 (15 years of age) exhibited increased exposure to THRX-651540, similar to the subject in Cohort 3, with telavancin exposure comparable to other subjects within Cohort 1. Previous findings in adults suggest there is significant variability in plasma THRX-651540 levels between individual subjects following telavancin administration (17). Safety of a single dose of telavancin was monitored by safety labs the day following the infusion as well as recording AEs experienced by the subjects. Most AEs experienced following telavancin administration were expected AEs associated with telavancin use (12). While two subjects experienced laboratory abnormalities that were resolved by the end of the follow-up period, common concerns associated with renal dysfunction with telavancin use in adults, including elevated serum creatinine and hypokalemia, were not observed in this brief study with only a single dose administered. It is possible that this was not observed in this pediatric study due to a small sample size, reduced exposure observed in children versus adults, or the fact that this study was a single dose of telavancin rather than a typical therapeutic multi-dose regimen. Infusion rate-dependent AEs, including flushing syndrome, have been reported with vancomycin (22), but there is limited data on infusion rate-dependent AEs with telavancin administration. Because of structural similarity and cross-reactivity between vancomycin and telavancin, there is a potential for similar infusion rate-dependent reactions with telavancin if administered incorrectly (23). A single 10 mg/kg infusion of telavancin was administered over an hour for this study according to dosing instructions for patients with normal renal function (12). Although there were no infusion reactions reported for this study, this is a potential area of investigation for future studies.

Studies in adults have demonstrated altered PK in obese patients, including a higher volume of distribution in patients with a BMI >40 kg/m^2^, making total body weight-based dosing less than ideal (24, 25). In this study, there were no participants with an obese BMI ≥30 kg/m^2^, and therefore these differences were not observed in any cohort. There were five participants considered overweight by BMI (25–29.9 kg/m^2^), and no notable differences in PK were observed between these participants and others within the same age cohort. As with adults, obesity should be considered as an additional potential variable in any future studies investigating telavancin PK and efficacy in pediatric populations.

This is the first study demonstrating the PK and short-term safety of telavancin in pediatric subjects. A previous report highlighted five different courses of telavancin in three adolescent cystic fibrosis patients with documented MRSA infection and previous adverse reactions to other antibiotics. One subject could not tolerate telavancin due to hypersensitivity and discontinued following a single dose, but the two other subjects tolerated 8.7–10 mg/kg daily doses of telavancin for up to 3 weeks with no significant safety concerns observed. Across all five courses of treatment, serum creatinine remained stable (16).

Telavancin, which is indicated for the treatment of cSSSI and HABP/VABP in adults, has demonstrated activity against multiple drug-resistant and -intermediate bacterial strains, including MRSA, VISA, enterococci*,* β-hemolytic streptococci, and viridan group streptococci (7, 8, 26). Telavancin has also demonstrated biofilm penetration and prevention as well as bactericidal efficacy against biofilm-resident pathogens in both *in vitro* models and *in vivo* animal models (27). These advantages present telavancin, a vancomycin derivative, as a viable option in the case of antibiotic failure in the treatment of certain Gram-positive bacterial infections. In children, while third- or fourth-generation antibiotics are typically used as first-line treatment for bacterial infection, other antibiotics, including vancomycin and ceftaroline, are used in cases of drug-resistant bacterial infections such as MRSA (2, 28). Recent studies have suggested that novel lipoglycopeptide antibiotics may be superior to vancomycin therapy in the treatment of soft tissue infections in adults and children (29, 30). The use of several major classes of antibiotic treatments, including fluoroquinolones and tetracyclines, is limited in pediatric patients of certain ages due to the potential for musculoskeletal adverse effects (2, 3). Furthermore, there are sparse data for the PK and pharmacodynamics of many antibiotic therapies in children, increasing the risk of incorrect dosing, leading to the potential for toxicity, lack of efficacy, and drug-resistance (2). Currently, tedizolid and oritavancin are under investigation for the treatment of certain bacterial infections in children (31, 32). The results of this study suggest that pediatric subjects 2–17 years of age experience reduced exposure to telavancin following a single 10 mg/kg IV infusion when compared with adults.

This study has some limitations. The study was designed to determine PK and short-term safety in children greater than 1 year of age. During the study, the US FDA concluded that the use of telavancin therapy in patients <6 years of age is unlikely due to risk of nephrotoxicity early in renal development, compared with other non-glycopeptide antibiotics currently in use for MRSA infections in young children, and released the sponsor from requirements for post-adult-marketing investigations for younger children and infants. Therefore, the study was closed prior to completing enrollment in Cohort 3 (2–5 years) and prior to enrolling any subjects in Cohort 4 (1 to <2 years). Cohort 3 consisted of a single 2-year-old subject with a potentially confounding medical condition, and therefore, no conclusions can be drawn regarding PK and safety in this age group. This study included the administration of a single dose of telavancin to generate PK data for subsequent clinical trials of telavancin in pediatrics. Additional studies are warranted to fully assess the long-term safety and PK of multiple doses of telavancin in pediatric subjects.

### Conclusions

This is the first clinical study to evaluate the short-term PK and safety of single-dose telavancin, a novel antibiotic with dual mechanisms of antimicrobial activity, in subjects 2–17 years of age. Results document reduced telavancin exposure in pediatric subjects compared with adults receiving the same mg/kg dose. Therefore, higher doses, not yet defined, will be required to achieve the exposures achieved in adults that have been associated with successful clinical and microbiologic outcomes. Future studies are warranted to adequately examine the PK, safety, and efficacy of therapeutic telavancin dosing regimens in pediatric subjects.

## MATERIALS AND METHODS

### Patient population

This was a multi-center (seven sites), open-label study investigating the PK and safety of a single dose of telavancin in pediatric subjects >12months to <17 years of age (Clinicaltrials.gov #NCT02013141). All study procedures were reviewed and approved by the respective Institutional Review Board for each study site. Prior to any study procedures, written informed consent from a parent or legal guardian and assent (if appropriate for older patients) were obtained. The patient population consisted of subjects aged >12 months to <17 years of age who required systemic antibiotic therapy for the treatment or prevention of known or suspected bacterial infection. Subjects were enrolled into four cohorts stratified by age: adolescents (12–17 years of age), older children (6–11 years of age), younger children (2–5 years of age), and infants (1–2 years of age). Subjects with an estimated creatinine clearance <50 mL/min/1.73 m^2^, clinically significant abnormal laboratory values, abnormal electrocardiogram (ECG), or those requiring monitoring of coagulation, or those receiving vancomycin (due to assay interference) were excluded from the study. The study consisted of a screening period followed by a treatment period and a safety follow-up period through Day 8 ± 1 day.

### Sample size

The standard regulatory requirement for sample size in pediatric studies is to prospectively power to target a 95% CI within 60% and 140% of the geometric mean estimates of clearance (CL) and volume of distribution (Vdss) in each pediatric age subgroup with at least 80% power (33, 34). Using the most conservative estimate of SD (0.284 for CL derived from the population PK model), a sample size of 7 per age subgroup was deemed sufficient to meet the aforementioned requirements with at least 80% power. Subjects were stratified by age (Cohort 1: 12–17 years, Cohort 2: 6–11 years, Cohort 3: 2–5 years, and Cohort 4: 1–2 years). In anticipation of attrition or missing samples, eight subjects were to be enrolled in each age subgroup with six in Cohort 4, which would result in a total of approximately 30 male and female infants, children, and adolescents. Sample size for each pediatric age subgroup was determined based on variability in telavancin PK parameters from adult data. Data from all Phase 1 telavancin studies (eight studies, which included a total of 236 healthy subjects without infection) conducted by Theravance and Cumberland Pharmaceuticals Inc. were used to estimate variability based on both noncompartmental analysis and population PK modeling.

### Drug administration and PK sampling

Each subject received a single, 10 mg/kg IV dose of telavancin infused over approximately 60 min. This dose was selected for the first study in pediatric populations based on the approved clinically effective dose of telavancin administered in adult subjects with normal renal function (12). The drug was infused over 60 min on Day 1. Blood samples were collected at 1, 1.5, 2, 6, 12, and 24 h following the start of infusion for PK analysis. The collected plasma was analyzed for both telavancin and its primary metabolite, THRX-651540.

### PK analysis and modeling

All subjects who received a dose of telavancin and provided PK data from a minimum of one post-dose sample were included in the PK population. Subjects with missing early PK samples were included in the PK population for plasma concentrations at each collected time point, but they were excluded from the PK modeling. Noncompartmental PK analysis and descriptive statistics were calculated from plasma concentrations of telavancin and THRX-651540 at each time point. The primary endpoints calculated from the plasma concentration-time data were maximum observed plasma concentration (*C*_max_), time to reach maximum plasma concentration (*T*_max_), area under the plasma concentration versus time curve from time 0 to the last sample with measurable analyte concentration (AUC_0-last_), area under the concentration versus time curve extrapolated to infinity (AUC_inf_), and terminal elimination half-life (*T*_1/2_). CL and apparent steady-state volume of distribution (Vss) were also calculated from the plasma concentration-time data. PK parameters were calculated using noncompartmental analysis with Phoenix WinNonlin v8.1 (Certara, Princeton, NJ, USA).

For calculation of mean concentrations at each time point and generation for mean concentration versus time profiles, all values below the limit of quantification (BLQ) were set to 0. When an individual value BLQ result fell between two quantifiable values, it was treated as missing data. For the NCA, actual infusion durations and actual sampling times were used. Infusion duration was calculated as the difference between the start of infusion time and the end of infusion time. Actual sampling times were calculated as the difference between the start of infusion time and the sampling time.

For PK analysis and individual concentration versus time plots, a concentration that was BLQ was assigned a value of 0, if it occurred in a profile before the first measurable concentration and was set to missing thereafter, if two BLQ values occurred in succession after *C*_max_, the profile was deemed to have terminated at the first BLQ value, and any subsequent concentrations were omitted from PK calculations.

### Safety

Safety was monitored during both the screening and treatment periods by physical exams, vital signs, urinalysis, and clinical laboratory measures (complete blood count with differential, serum chemistry, creatinine, and liver enzymes); AEs and concomitant medications were recorded from screening through follow-up on Day 8 (± 1 day). Medical and medication history and demographics were collected at screening. Vital signs (heart rate, blood pressure, respiratory rate, and body temperature) were collected at screening, Days 1 and 2 of the study. To monitor potential cardiac abnormalities, 12-lead ECGs were conducted at rest at both screenings and between 1.5 and 2.5 h following the start of telavancin infusion.

## CLINICAL TRIAL REGISTRATION

Clinicaltrials.gov Registration Number: NCT02013141.

## SUPPLEMENTAL MATERIAL

Table S1 aac.00987-23-s0001.docx Summary of select laboratory values by cohort before and after telavancin dosing

ASM does not own the copyrights to Supplemental Material that may be linked to, or accessed through, an article. The authors have granted ASM a non-exclusive, world-wide license to publish the Supplemental Material files. Please contact the corresponding author directly for reuse.
