# Supplementary material for: Pharmacokinetics and safety of a single dose of telavancin in pediatric subjects 2–17 years of age
Source: Antimicrob Agents Chemother. 2023 Oct 10;67(11):e00987-23. doi: 10.1128/aac.00987-23 (PMC10649008; doi:10.1128/aac.00987-23)
Supplement: Table S1 — Summary of select laboratory values by cohort before and after telavancin dosing [file aac.00987-23-s0001.docx]

**SUPPLEMENTARY MATERIAL**

| **Table S1 Summary of select laboratory values by cohort pre- and post-telavancin** | | | | |
| --- | --- | --- | --- | --- |
| **Laboratory Value** | **Time Point** | **Cohort 1 (12 to 17 years)**  **N = 14^a^** | **Cohort 2**  **(6 to 11 years)**  **N = 7^b^** | **Cohort 3**  **(2 to 5 years)**  **N = 1** |
| Hemoglobin, g/dL | Pre-dose | 12.2 ± 1.7 | 11.7 ± 1.4 | 10.0 ± 0.0 |
|  | Post-dose | 12.1 ± 1.4 | 11.9 ± 1.4 | 10.3 ± 0.0 |
| Leukocytes, x10^9^/L | Pre-dose | 10.3 ± 3.1 | 8.3 ± 3.0 | 4.6 ± 0.0 |
|  | Post-dose | 7.3 ± 2.0 | 6.3 ± 1.3 | 7.5 ± 0.0 |
| Platelets, x10^9^/L | Pre-dose | 226 ± 69.1 | 267 ± 73.6 | 148 ± 0.0 |
|  | Post-dose | 236 ± 85.9 | 289 ± 105.5 | 195 ± 0.0 |
| Creatinine, mg/dL | Pre-dose | 0.6 ± 0.2 | 0.4± 0.1 | 0.2 ± 0.0 |
|  | Post-dose | 1.0 ± 1.1 | 0.4 ± 0.1 | 0.2 ± 0.0 |
| Total bilirubin, mg/dL | Pre-dose | 0.6 ± 0.3 | 0.6 ± 0.5 | 0.4 ± 0.0 |
|  | Post-dose | 0.3 ± 0.2 | 0.5 ± 0.3 | 0.4 ± 0.0 |
| Aspartate aminotransferase, IU/L | Pre-dose | 50.9 ± 56.2 | 41.6 ± 26.0 | 41.0 ± 0.0 |
|  | Post-dose | 72.8 ± 72.0 | 46.7 ± 40.7 | 37.0 ± 0.0 |
| Alanine aminotransferase, IU/L | Pre-dose | 25.9 ± 15.1 | 30.9 ± 12.2 | 72.0 ± 0.0 |
|  | Post-dose | 37.4 ± 24.2 | 36.8 ± 19.4 | 62.0 ± 0.0 |
| Lactate dehydrogenase, IU/L | Pre-dose | 361 ± 247 | 534± 150 | 477 ± 0.0 |
|  | Post-dose | 439 ± 270 | 588 ± 145 | 480 ± 0.0 |
| ^a^Only 9 subjects had post-dose laboratory markers collected in Cohort 1  ^b^Only 6 subjects had post-dose laboratory markers collected in Cohort 2 | | | | |
